# Why do knowledge workers choose knowledge hiding: the mediating role of perceived stress

**DOI:** 10.3389/fpsyg.2025.1708030

**Published:** 2026-01-02

**Authors:** Yina Bai, Yuxin Xiao

**Affiliations:** School of Business Administration, Liaoning Technical University, Huludao, China

**Keywords:** knowledge hiding, knowledge workers, perceived stress, task complexity, workplace ostracism

## Abstract

**Introduction:**

In today's knowledge-driven economy, knowledge workers are central to sustaining innovation, yet the phenomenon of Knowledge hiding—the deliberate withholding of requested knowledge—poses a serious challenge to collaboration and organizational performance. Drawing on conservation of resources theory, social exchange theory, and stress interaction theory, this study explores how Workplace Ostracism and task complexity influence knowledge hiding, emphasizing the mediating role of Perceived stress and the moderating role of Perceived pay fairness.

**Methods:**

Data were collected from 330 knowledge workers in Beijing through a two-wave survey combining online and offline questionnaires. Workplace Ostracism, task complexity, perceived stress, knowledge hiding, and perceived pay fairness were measured using validated scales. Hierarchical regression, confirmatory factor analysis, and bootstrapping techniques were applied to test direct, mediating, and moderating effects.

**Results:**

The results indicate that both Workplace Ostracism and Task complexity significantly and positively predict Knowledge hiding, with Perceived stress partially mediating these effects. Furthermore, Perceived pay fairness negatively moderates the relationship between Perceived stress and Knowledge hiding, as well as the indirect effects of Workplace Ostracism and Task complexity via Perceived stress. Specifically, high levels of Perceived pay fairness weaken these positive associations, whereas low levels exacerbate them.

**Discussion:**

This study makes three key contributions. First, it enriches the literature on Knowledge hiding by introducing a stress-perception perspective and highlighting Perceived stress as a central explanatory mechanism. Second, it extends conservation of resources theory and social exchange theory by identifying Perceived pay fairness as a critical boundary condition. Third, it offers actionable guidance for organizations, suggesting that enhancing compensation fairness, fostering inclusion, and managing task demands can mitigate stress-induced Knowledge hiding and promote effective knowledge sharing.

## 1 Introduction

In the era of the knowledge economy, knowledge serves as a fundamental engine of socioeconomic advancement. As the principal custodians and disseminators of knowledge, knowledge workers play an indispensable role in this process (Huo et al., 2016). Accordingly, organizations have made substantial investments to foster knowledge sharing among employees and to enhance the circulation of knowledge both within organizational boundaries and across external networks. Despite the recognized importance of knowledge sharing, the complexity of organizational environments, and the scarcity of knowledge resources often hinder its effective implementation, leaving overall levels of knowledge exchange relatively low (Yao et al., 2023). For instance, a survey conducted by Canada's Globe and Mail with more than 1,700 employees reported that nearly three-quarters of respondents engaged in knowledge hiding behaviors, such as reluctance to share expertise or withholding useful information when approached by colleagues. A similar pattern emerges in China, where (Peng 2012) found that 46% of knowledge workers displayed tendencies toward knowledge concealment. These findings highlight the pervasive phenomenon of knowledge hiding—defined as the intentional withholding or concealment of requested knowledge for specific purposes (Connelly et al., 2012). Such behaviors have been shown to undermine employee creativity, strain interpersonal relationships, weaken team performance, and lead to broader organizational inefficiencies (Jeong et al., 2022; Iftikhar, 2025). Accordingly, examining the underlying mechanisms of knowledge hiding and identifying strategies to mitigate it hold substantial theoretical significance and practical implications for advancing knowledge flows and enhancing organizational performance.

Given the prevalence of knowledge hiding and its detrimental effects on organizational outcomes, scholars have devoted considerable attention to identifying its antecedents. Prior research has demonstrated that job task characteristics, leadership styles, and unfavorable organizational attributes can all shape employees' propensity to conceal knowledge to varying degrees (Abubakar et al., 2019; Venz and Nesher Shoshan, 2022; Khalid and Hussain, 2025; Shen et al., 2025).

In practice, however, modern organizational contexts add further complexity to this phenomenon. For instance, in China, the “996” work schedule (working from 9 a.m. to 9 p.m., 6 days a week) has become prevalent in many enterprises, reflecting the intensification of work demands. Under such conditions, workplace stress has emerged as a pervasive challenge, with escalating “internal competition” constituting an indisputable reality for organizational members, particularly for knowledge workers. The sheer volume of tasks and limited time resources often create acute pressure, which in turn influences behavioral choices. Consequently, recent studies have begun to explore how different forms of pressure—such as time pressure, role pressure, and performance pressure—affect employees' engagement in knowledge sharing, silence, and concealment (Zhao and Jiang, 2022). Scholars contend that when employees experience stress, they often adopt protective strategies to conserve resources and avoid a downward spiral of continuous resource depletion, which ultimately leads to knowledge concealment (Jeong et al., 2022; Liang and Wang, 2022; Liu et al., 2025). The cognitive appraisal theory of stress conceptualizes stress formation as a dynamic process of individual cognition and appraisal arising from the interaction between the individual and the environment (Lazarus and Folkman, 1984; Mitchell et al., 2019). Stressors of varying nature exert differential effects on employees' learning motivation and performance, prompting individuals to evaluate the stress they encounter and to adopt either adaptive or maladaptive coping strategies accordingly (Lepine et al., 2005).

Although prior research has begun to investigate the relationship between stressful contexts and Knowledge hiding from the perspective of cost avoidance, the literature has primarily emphasized the impact of distinct stressor types. For example, (Yang et al. 2021) demonstrated that both challenge and hindrance stressors significantly foster Knowledge hiding, while (Liang and Wang 2022) further confirmed the positive influence of hindrance stressors. However, limited scholarly attention has been directed toward clarifying the essence of hindrance stressors—particularly the question of what specifically constitutes hindrance stressors for knowledge workers. This gap restricts the academic community's ability to fully comprehend how workplace stress arises for knowledge workers and how it subsequently translates into Knowledge hiding. Emerging studies suggest that negative situational factors such as Task complexity and Workplace Ostracism intensify employee stress (Xia et al., 2021). Yet, these investigations tend to overlook the mediating role of Perceived stress in linking such situational factors with Knowledge hiding. Moreover, research in this domain lacks a systematic framework that explicates the sources of hindrance stress for knowledge workers and the developmental process through which Perceived stress evolves to shape Knowledge hiding behaviors. Importantly, the extant literature has largely conceptualized Perceived stress as either an independent or moderating variable, while its potential mediating role remains underexplored and thus warrants rigorous empirical validation.

Therefore, this study investigates the mechanisms of employee Knowledge hiding from the perspective of stress perception, with particular attention to the mediating role of Perceived stress in the relationship between hindrance stressors—such as Task complexity and Workplace Ostracism—and Knowledge hiding behaviors. By adopting this perspective, the study not only elucidates the processes underlying the formation of Knowledge hiding but also clarifies the antecedent conditions and transmission pathways through which stress influences knowledge workers. This approach contributes to advancing theoretical understanding of stress–knowledge dynamics while offering actionable implications for organizational interventions aimed at mitigating Knowledge hiding and promoting effective knowledge sharing.

In addition, perceptions of Perceived pay fairness—a reflection of organizational management practices—are internalized within employees' daily work routines. These perceptions shape interpersonal and organizational interactions, ultimately influencing individual performance outcomes (Barber and Simmering, 2003). From the perspective of conservation of resources theory, compensation systems that fulfill employees' needs and foster perceptions of fairness constitute valuable resources, which can complement or substitute for other job resources and, in turn, shape employees' knowledge behaviors (Tremblay et al., 2000; Hobfoll et al., 1990). However, few studies have examined whether Perceived pay fairness moderates the relationship between Perceived stress and Knowledge hiding. Addressing this gap, the present study incorporates Perceived pay fairness into the research model. This approach clarifies both the mediating mechanisms and the boundary conditions through which hindrance stressors influence Knowledge hiding, thereby advancing theoretical understanding of stress–knowledge dynamics and providing practical guidance for organizations seeking to mitigate Knowledge hiding and promote effective knowledge circulation.

## 2 Theoretical background and hypothesis development

### 2.1 The relationship between Workplace Ostracism and knowledge hiding

Workplace ostracism is defined as employees' perceptions of being marginalized, ignored, or dismissed by colleagues (Ferris et al., 2008; Li and Li, 2025; Indhumathi et al., 2025). With the intensification of organizational change and the growing complexity of workplace dynamics, Workplace ostracism has become increasingly prevalent. In China, for instance, more than 70% of knowledge workers report experiencing Workplace ostracism (Su et al., 2021; Liu et al., 2011; Peng, 2013). Prior research demonstrates that ostracism undermines fundamental psychological needs, including self-esteem, belongingness, and control. It provokes intense negative emotions, impairs physiological functioning, including immune responses, and generates adverse organizational outcomes by heightening turnover intentions and deviant behaviors while simultaneously undermining performance (Williams, 2007; Howard et al., 2020; Sharma and Dhar, 2024). Drawing on conservation of resources theory, individuals instinctively reduce resource expenditure when confronted with resource threats to safeguard their existing psychological and cognitive reserves. When employees frequently encounter Ostracismary experiences in the workplace, they perceive diminished social support and a devaluation of emotional worth, which in turn activates self-protective mechanisms. As a result, employees may deliberately engage in Knowledge hiding to minimize exposure to social risks and avoid further resource depletion in interpersonal exchanges. Thus, Workplace ostracism restricts employees' access to task-related resources and consequently amplifies Knowledge hiding (Li, 2021; Srivastava and Dhir, 2024).

In parallel, social exchange theory conceptualizes interpersonal interactions as a process of resource exchange governed by cost–benefit considerations, reciprocity norms, and trust (Taylor et al., 2012; Xi et al., 2018). Workplace ostracism disrupts the norm of reciprocity in social relations. Following Ostracismary experiences, employees often perceive colleagues or the organization as untrustworthy, thereby fostering negative emotions, perceived threats to fundamental needs, and heightened psychological distress. In response, they are likely to withdraw from knowledge-sharing and engage in defensive self-protection strategies, such as refusing others' requests for knowledge and adopting Knowledge hiding behaviors (Gao and He, 2019; Srivastava et al., 2024). Accordingly, the following hypothesis is proposed.

**H1:** Workplace ostracism is positively associated with Knowledge hiding behavior among knowledge workers.

### 2.2 The relationship between task complexity and knowledge hiding

Knowledge workers typically engage in tasks characterized by high levels of autonomy, complexity, interdependence, uncertainty, and creativity (Rasheed and Pitafi, 2024). Prior research demonstrates that distinct task attributes exert heterogeneous effects on employee behavior, with task interdependence, conflict, and autonomy each influencing Knowledge hiding to varying extents (Fu et al., 2020; Salimzadeh et al., 2024; Rasheed and Pitafi, 2024). In the context of organizational digitalization and intelligent transformation, knowledge workers are increasingly confronted with dynamic and multifaceted task environments, where work demands have grown progressively more intricate.

Task complexity is generally defined as the degree of difficulty associated with task execution (Morgeson and Humphrey, 2006; Piazza et al., 2019). From the perspective of conservation of resources theory, individuals faced with resource threats tend to adopt resource-conserving strategies to prevent mental exhaustion. High Task complexity entails challenging assignments that demand employees deploy multiple advanced skills, thereby imposing greater cognitive and psychological burdens. Consequently, Task complexity is widely regarded as a hindrance stressor capable of inducing negative psychological states, including tension, stress, and cognitive overload, all of which deplete cognitive and emotional resources (Wood, 1986; Simon, 2019). To mitigate such depletion, employees may resort to Knowledge hiding as a self-protective strategy, conserving resources to cope with core task requirements.

In addition, social exchange theory offers a complementary perspective. Elevated Task complexity compels knowledge workers to perform more actions and process larger volumes of information, requiring substantial investments of cognitive, knowledge, and time resources. When employees perceive that these additional efforts are insufficiently reciprocated or rewarded, the norm of reciprocity is disrupted, prompting individuals to withhold knowledge as a means of rebalancing the exchange relationship.

**H2:** Task complexity is positively associated with Knowledge hiding behavior among knowledge workers.

### 2.3 The moderating role of perceived stress

Perceived stress refers to employees' feelings of uncertainty and threat when performing work tasks and striving to meet organizational performance standards, typically manifesting as physical and psychological tension and anxiety (Hore-Lacy et al., 2024; Cook et al., 2024). In the context of intensifying market competition, the proliferation of demanding work schedules such as “996” or “997” has made occupational stress a pervasive phenomenon in the workplace (Tandler et al., 2020).

From the perspective of social exchange theory, strong exchange relationships between employees and organizations encourage individuals to engage in extra-role behaviors. When such relationships are disrupted, however, employees tend to reduce their discretionary contributions in line with principles of reciprocity and equivalent exchange (Taylor et al., 2012). Workplace Ostracism and perceived job insecurity represent clear signals that the organization has undermined its established exchange relationship with employees. Under such circumstances, knowledge workers—concerned about their career prospects and motivated by resource conservation—lower their evaluations of organizational support and commitment. Consequently, their willingness to engage in knowledge sharing diminishes. Specifically, Workplace Ostracism heightens negative experiences and pessimistic perceptions, which, after cognitive processing, reinforce sensitivity to one's internal organizational status. This heightened awareness of status vulnerability translates into Perceived stress, leading employees to avoid extra-role behaviors—particularly risk-laden activities such as knowledge sharing—due to concerns over instability and loss of standing (Sharma and Dhar, 2024).

Conservation of resources theory further illuminates this process. Its central premise is that individuals are motivated to conserve, protect, and acquire valued resources (Hobfoll et al., 1990). When resources are threatened or depleted, individuals experience stress and strive to take preventive actions to avoid a spiral of escalating loss (Xiao et al., 2023). Compared with non-knowledge workers, knowledge workers rely primarily on intellectual rather than manual labor and attach heightened value to knowledge resources. Workplace Ostracism undermines their access to material support and relational resources, both of which are critical for achieving work and career goals. In response, knowledge workers may adopt defensive and even destructive behaviors—such as Knowledge hiding—to preserve remaining core resources and avoid further losses.

Based on the above reasoning, this study advances the following hypotheses:

**H3:** Perceived stress mediates the positive relationship between Workplace Ostracism and Knowledge hiding behavior.

Workplace stress exerts subtle yet targeted threats to employees' emotional cognition and work attitudes, with its effects shaped by both the dynamics of employment relationships and individual differences (Cook et al., 2024). In the context of workplace “involution,” the traditional economic exchange—where job responsibilities were narrowly defined and contribution expectations remained low—has been disrupted. Employees now face heightened stress experiences, as its persistent and invisible nature threatens not only physical and psychological health but also work-related attitudes (Tandler et al., 2020).

From a social exchange theory perspective, organizations provide employees with resources while simultaneously imposing reciprocal expectations (Taylor et al., 2012). On one hand, such resources satisfy psychological and material needs and enhance career growth, thereby stimulating work motivation. On the other hand, meeting heightened expectations can become a significant source of pressure. Knowledge workers, as critical carriers of intellectual capital and executors of organizational innovation, are subject to particularly high expectations for creative contributions. In pursuit of innovation goals, they expend substantial emotional and cognitive resources, often experiencing emotional exhaustion (Bakker et al., 2014). Moreover, the increasing Task complexity of knowledge-intensive work requires greater cognitive effort to address multifaceted workplace challenges. When employees perceive their contributions as inadequately reciprocated, the reciprocity balance of social exchange is disrupted, leading to feelings of stress and pressure. Consequently, knowledge workers may engage in Knowledge hiding behaviors as a means of rebalancing the exchange relationship.

Furthermore, hindrance stressors such as high Task complexity, heavy workloads, and procedural barriers consume significant emotional and mental resources while exacerbating work–family conflict and fostering negative emotions. These adverse sentiments, coupled with intensified workplace competition, further reinforce employees' propensity toward Knowledge hiding.

Based on the above reasoning, the following hypotheses are proposed:

**H4:** Perceived stress mediates the positive relationship between Task complexity and Knowledge hiding.

### 2.4 The moderating role of perceived pay fairness

Fairness has long been a central concern in organizational management. Perceived organizational unfairness may undermine employee motivation, reduce work efficiency, or even trigger talent loss, ultimately threatening the survival and development of organizations (Wu and Liu, 2009; Mohrenweiser and Pfeifer, 2023). Among various dimensions of fairness, Perceived pay fairness refers to employees' subjective evaluation of whether compensation outcomes are reasonable, commensurate with their contributions, and distributed fairly—often assessed through comparisons of their effort–reward ratio with personal experience or that of others. Such perceptions exert a particularly strong influence on employees' work-related attitudes and behaviors (Strauss et al., 2025; Zhang L. et al., 2025).

Existing research suggests that Perceived pay fairness reflects organizational recognition and support for employee contributions. It satisfies employees' psychological needs, strengthens their sense of responsibility and mission, and encourages them to engage in extra-role behaviors such as knowledge sharing (Barber and Simmering, 2003; Kamilçelebi and Burger, 2025). In essence, Perceived pay fairness represents an equitable and supportive organizational environment that enhances employees' trust in the organization. Employees reciprocate this trust by displaying more positive attitudes and behaviors.

The Stress Interaction Theory emphasizes that stress not only arises from external environmental threats or resource losses but also from individuals' interactive assessments of stressors. Specifically, individuals tend to re-evaluate stressors based on their psychological resources, social support, and other factors when confronted with stress, which subsequently influences their behavioral responses (Lazarus and Folkman, 1984). This provides a reasonable explanatory framework for understanding how workplace Ostracism and task complexity are transformed into stress through cognitive appraisal, which ultimately affects the knowledge hiding behavior of knowledge workers.

From the perspective of the Stress Interaction Theory, perceptions of pay fairness are not only a type of external resource but also an important factor that moderates employees' cognitive and emotional responses. When employees perceive that the organization distributes compensation fairly, they view the organization as a supportive and psychologically safe environment, which provides them with more psychological resources. In the framework of stress interaction, a high perception of pay fairness can alleviate the stress caused by workplace Ostracism or task complexity. This occurs because employees re-evaluate stressors and perceive them as acceptable challenges rather than threats, thereby reducing the occurrence of knowledge hiding behavior.

Specifically, the perception of pay fairness enhances employees‘ trust in the organization and, by improving psychological safety and organizational support, increases their commitment to organizational goals. When employees feel that their contributions are fairly compensated by the organization, they tend to align their personal goals with the organization's objectives and invest more effort. In this context, employees' negative emotions and stress are transformed into reciprocal resources, which reduces the knowledge hiding mindset triggered by stress perception.

Based on the above reasoning, the following hypothesis is proposed:

**H5**: Perceived pay fairness moderates the positive relationship between Perceived stress and Knowledge hiding, such that the relationship is weaker when perceived pay fairness is high.

Complex work task goals mean that employees need to consume more knowledge resources, time resources, cognitive resources, and so on. A high level of perceived pay fairness, however, gives employees hope for future promotions and salary increases, thereby enhancing their sense of self-efficacy and other psychological resources. According to the Stress Interaction Theory, this stressor is not only a threat faced by individuals, but also the result of the interaction between their cognitive appraisal of the stressor and their internal resources. Therefore, the workplace stress caused by complex work task goals is not simply seen as a resource threat, but is transformed, from the perspective of stress interaction, into an expectation of future resource returns. In other words, overcoming this pressure not only enhances psychological satisfaction but may also lead to financial growth and career advancement. In this context, complex work tasks are not merely a requirement for knowledge workers to contribute to the team or others; they also represent an opportunity and a way to meet personal economic needs through work pressure and to strive for future development and growth, thereby reducing negative behaviors that could affect the organization.

Additionally, workplace Ostracism damages the interpersonal relationship resources that employees value, leaving their needs for social belonging and self-esteem unmet. However, for employees with a high perception of pay fairness, the organizational support, psychological safety, and other resources they gain from the organization enable them, through the lens of stress interaction, to transform the stress caused by workplace Ostracism into a positive coping motivation. According to the Stress Interaction Theory, individuals do not passively endure stressors but rather, through cognitive appraisal, transform them into manageable and controllable challenges. In this situation, knowledge workers, based on their expectation of positive feedback, may transform the stress caused by workplace Ostracism into a driving force to repair their interpersonal networks. They might actively change the way they communicate and interact with colleagues, share their knowledge, skills, and experiences, and increase their popularity within the employee group (Dong and Zhang, 2025). This transformation of stress not only helps them reintegrate into the employee group but also aids in their rapid recovery from high-pressure states. Based on the above reasoning, the following hypotheses are proposed:

**H6a:** Perceived pay fairness moderates the relationship between Workplace Ostracism and Knowledge hiding, such that the positive relationship is weaker when perceived pay fairness is high.
**H6b:** Perceived pay fairness moderates the relationship between Task complexity and Knowledge hiding, such that the positive relationship is weaker when perceived pay fairness is high.

**Figure 1:**
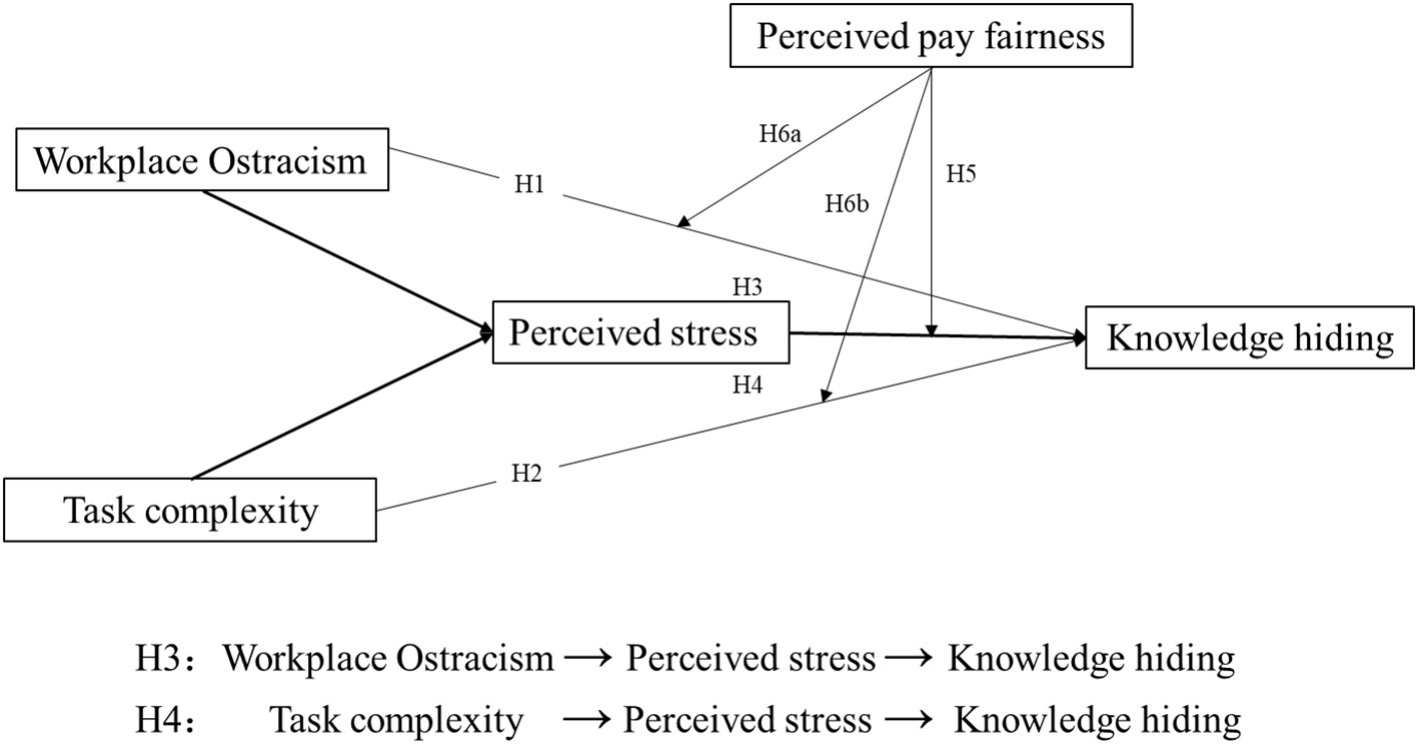
Theoretical model.

The theoretical model is shown in Figure 1.

## 3 Methodology

### 3.1 Sample and data collection procedure

This study employed a mixed-method survey approach, integrating online questionnaires (administered via the Question Star platform) with offline paper-based surveys. Participants were provided with appropriate compensation to incentivize their participation. The sample data was collected from knowledge workers across 10 companies spanning a range of industries, including internet, finance, consulting, food processing, real estate, and apparel manufacturing sectors, all based in Beijing. Among these 10 companies, 3 are European and American enterprises, while 7 are domestic Chinese firms. Beijing was intentionally chosen as the survey location for two primary reasons. First, as China's political and economic center, Beijing is known for its fast-paced work environment, heavy workloads, and intense workplace competition—factors that align with the high-pressure context central to this study. Second, Beijing hosts several knowledge-intensive industrial clusters, such as Zhongguancun Science Park, Financial Street, and the Central Business District, making it a representative hub for knowledge workers relevant to the study's focus.

To mitigate the potential impact of common method bias, all questionnaires were completed anonymously. Following the approach of (Cook et al. 2024), data was collected in two separate time periods, with a two-month interval between the two rounds of data collection. In Phase One, measures of Workplace Ostracism, Task complexity, Perceived stress, and control variables were collected. Online questionnaires were distributed through MBA programs, training seminars, alumni networks, and personal contacts, yielding 350 valid responses. Additionally, 100 paper based surveys were distributed through on-site visits to internet firms in Zhongguancun. In Phase Two, using the same distribution channels, data on Perceived pay fairness and Knowledge hiding were collected. A total of 387 questionnaires were returned across both phases. After matching responses by company identifiers and excluding invalid cases (e.g., uniform response patterns, missing data, or incomplete submissions), 330 valid questionnaires remained, resulting in a final effective response rate of 73.3%.

The demographic characteristics of the sample are as follows. By gender, 161 were male (48.8%) and 169 were female (51.2%). With respect to age, 92 respondents (27.9%) were aged 25 and below, 79 (23.9%) were aged 26–35, 71 (21.5%) were aged 36–45, and 88 (26.7%) were aged 46 and above. Regarding educational attainment, the majority held a bachelor's degree (131; 39.7%) or a master's degree (75; 22.7%). In terms of work experience, 99 participants (30.0%) reported 1–3 years, while 159 (48.2%) reported 7 or more years. Finally, by organizational level, regular employees represented the largest proportion (164; 49.7%), followed by middle managers (63; 19.1%).

### 3.2 Measurement of variables

All study variables were measured using well-established scales that have demonstrated reliability and validity in both domestic and international research. Unless otherwise specified, all items were rated on a five-point Likert scale ranging from 1 (“strongly disagree”) to 5 (“strongly agree”).

Workplace Ostracism was measured using the 10-item scale developed by (Ferris et al. 2008). A sample item is “At work, I feel ignored.” The scale demonstrated satisfactory reliability and validity (Cronbach's α = 0.932, CR = 0.933, AVE = 0.581).

Task complexity was assessed with a three-item scale developed by (Wood 1986). A sample item is “I need to process a large amount of information and numerous steps in my work.” The scale demonstrated acceptable reliability (Cronbach's α = 0.788, CR = 0.790, AVE = 0.558).

Perceived stress was measured using the 10-item unidimensional scale developed by (Cohen et al. 1983). A sample item is “I feel uneasy when unexpected events occur.” The scale demonstrated high internal consistency (Cronbach's α = 0.950, CR = 0.950, AVE = 0.657).

Knowledge hiding was assessed using the 12-item scale developed by (Connelly et al. 2012), which captures three dimensions: rationalized hiding, evasive hiding, and feigned ignorance. The scale demonstrated acceptable reliability (Cronbach's α = 0.868, CR = 0.872, AVE = 0.375).

Perceived pay fairness was measured using a five-item unidimensional scale developed by (Shaw and Gupta 2001). This scale, adapted to the characteristics of knowledge workers in this study context, evaluates perceptions of pay fairness from both internal and external perspectives. Reliability was acceptable (Cronbach's α = 0.850, CR = 0.851, AVE = 0.536).

Control variables included gender, age, education level, tenure, and job level, in order to rigorously examine the focal relationships among Workplace Ostracism, Task complexity, Perceived stress, Perceived pay fairness, and Knowledge hiding.

## 4 Data analysis and empirical testing

### 4.1 Common method bias

To address the potential concern of Common Method Bias (CMB) inherent in self-reported survey data, both procedural and statistical remedies were employed. First, procedural safeguards were implemented: respondents were informed that the data would be used exclusively for academic research, with strict confidentiality and anonymous data collection, thereby encouraging candid responses. Moreover, the order of items was randomized to generate three different versions of the questionnaire, with one version distributed at each data collection wave to further mitigate CMB.

Second, the suitability of the measurement items for confirmatory factor analysis was examined. Results of the Kaiser–Meyer–Olkin (KMO) test (KMO = 0.930) and Bartlett's test of sphericity (*p* < 0.001) indicated that the data were appropriate for factor analysis.

Furthermore, Harman's single-factor test was conducted. The first principal component explained 24.81% of the variance, which is below the critical threshold of 40%, indicating that no single factor accounted for the majority of the variance. Finally, a single-factor confirmatory factor analysis was performed. The results (χ^2^/df = 5.904, RMSEA = 0.122, CFI = 0.515, SRMR = 0.130) demonstrated a significantly poorer fit than the hypothesized multi-factor measurement model, further supporting the absence of substantial CMB.

Taken together, these results indicate that Common Method Bias was not a serious concern in this study.

### 4.2 Reliability and validity analyses

The reliability and validity of the measurement scales were examined using SPSS 26.0 and Amos 26.0. Results indicated that Cronbach's α co efficients and Composite Reliability (CR) values for all constructs were above the recommended threshold of 0.70, indicating satisfactory internal consistency reliability. In addition, all standardized factor loadings derived from confirmatory factor analysis (CFA) exceeded 0.60, and the Average Variance Extracted (AVE) values of all constructs were above the 0.50 criterion, providing evidence for adequate convergent validity (Show in Table 1). Furthermore, the hypothesized five-factor measurement model fit the data significantly better than alternative models (χ^2^/df = 1.837, RMSEA = 0.05, CFI = 0.918, TLI = 0.913, SRMR = 0.051), indicating strong discriminant validity among the study variables. Collectively, these findings demonstrate that the measurement model possesses satisfactory reliability and validity.

**Table 1:** Confirmatory factor analysis result.

**Model**	**χ^2^**	**df**	**χ^2^/df**	**RMSEA**	**SRMR**	**CFI**	**TLI**
Five-factor model (WO + TC + PS + PP + KH)	1341.210	730	1.837	0.050	0.051	0.918	0.913
Four-factor model (WO + TC + PS + KH, PP)	1865.499	734	2.541	0.068	0.069	0.849	0.839
Three-factor model (WO + TC + PS, PP, KH)	2741.406	737	3.719	0.091	0.107	0.732	0.717
Two-factor model (WO + TC + PS, PP + KH)	3004.995	739	4.066	0.096	0.119	0.697	0.68
One-way model (WO, TC, PS, PP, KH)	4369.023	740	5.904	0.122	0.130	0.515	0.489

WO, Workplace Ostracism; TC, Task Complexity; PS, Perceived Stress; PP, Perceived Pay Fairness; KH, Knowledge Hiding.

### 4.3 Descriptive statistics and correlations

The descriptive statistics and correlation coefficients of all study variables are presented in Table 2, based on the 330 valid responses. As shown in Table 2, Task complexity was significantly and positively correlated with Knowledge hiding (r = 0.473, *p* < 0.01), Workplace Ostracism (r = 0.384, *p* < 0.01), and Perceived stress (r = 0.455, *p* < 0.01). These correlations are consistent with the theoretical framework and provide preliminary empirical support for the subsequent hypothesis testing.

**Table 2:** Means, standard deviations, and correlations between variable.

**Variant**	**1**	**2**	**3**	**4**	**5**	**6**	**7**	**8**	**9**	**10**
**1.Gender**	1									
**2.Age**	−0.096	1								
**3.Education**	−0.037	0.448^**^	1							
**4.Working years**	−0.09	0.905^**^	0.493^**^	1						
**5.Job level**	−0.063	0.523^**^	0.860^**^	0.579^**^	1					
**6.WO**	0.125^*^	−0.002	−0.057	0.011	−0.024	1				
**7.TC**	0.01	0.022	0.021	0.044	0.089	0.416^**^	1			
**8.PS**	0.041	−0.05	−0.09	−0.033	−0.045	0.415^**^	0.448^**^	1		
**9.KH**	0.001	−0.081	−0.161^**^	−0.069	−0.114^*^	0.384^**^	0.473^**^	0.455^**^	1	
**10.PP**	0.072	−0.045	−0.000	−0.037	0.036	−0.234^**^	−0.268^**^	−0.204^**^	−0.313^**^	1
**M**	1.512	2.470	2.188	2.939	2.939	3.27	3.276	3.024	3.268	2.648
**SD**	0.501	1.159	0.952	1.126	1.400	0.796	0.815	0.580	0.653	0.793

WO, Workplace Ostracism; TC, Task Complexity; PS, Perceived Stress; PP, Perceived Pay Fairness; KH, Knowledge Hiding. ^**^p < 0.01, ^*^p < 0.05.

### 4.4 Hypothesis testing

Hierarchical regression analysis was conducted to test the proposed hypotheses, and the results are summarized in Table 3. All explanatory variables were standardized prior to analysis. The VIF values for each model were below 2, indicating the absence of multicollinearity.

**Table 3:** Results of hierarchical regression analyses.

**Variables**	**Perceived stress**			**Knowledge hiding**		
	**Model 1**	**Model 2**	**Model 3**	**Model 4**	**Model 5**	**Model 6**
**Control variates**						
Gender	0.046	−0.001	−0.005	−0.051	−0.051	−0.010
Age	−0.052	0.03	−0.057	−0.031	−0.023	−0.022
Education	−0.120	−0.052	−0.163^*^	−0.084	−0.07	−0.098
Working years	0.044	0.019	0.049	0.022	0.017	0.014
Job lever	0.055	0.006	0.044	−0.016	−0.017	0.003
**Independent variable**						
Workplace ostracism		0.198^**^		0.180^**^	0.126^**^	0.151^**^
Task complexity		0.239^**^		0.310^**^	0.245^**^	0.248^**^
**Mediator variable**						
Perceived stress					0.274^**^	0.958^**^
**Moderator Variable**						
Perceived pay fairness						0.609^**^
**Interaction Effect**						
Perceived stress × Perceived Pay fairness						−0.261^**^
*R* ^2^	0.016	0.272	0.03	0.278	0.337	0.395
Δ*R*^2^	0.016	0.256	0.03	0.263	0.059	0.058
*F*	1.033	56.626^***^	1.995^*^	17.170^***^	20.353^***^	23.209^***^

Standardized coefficients (β) are reported. ^***^p < 0.001, ^**^p < 0.01, ^*^p < 0.05.

**Figure 2:**
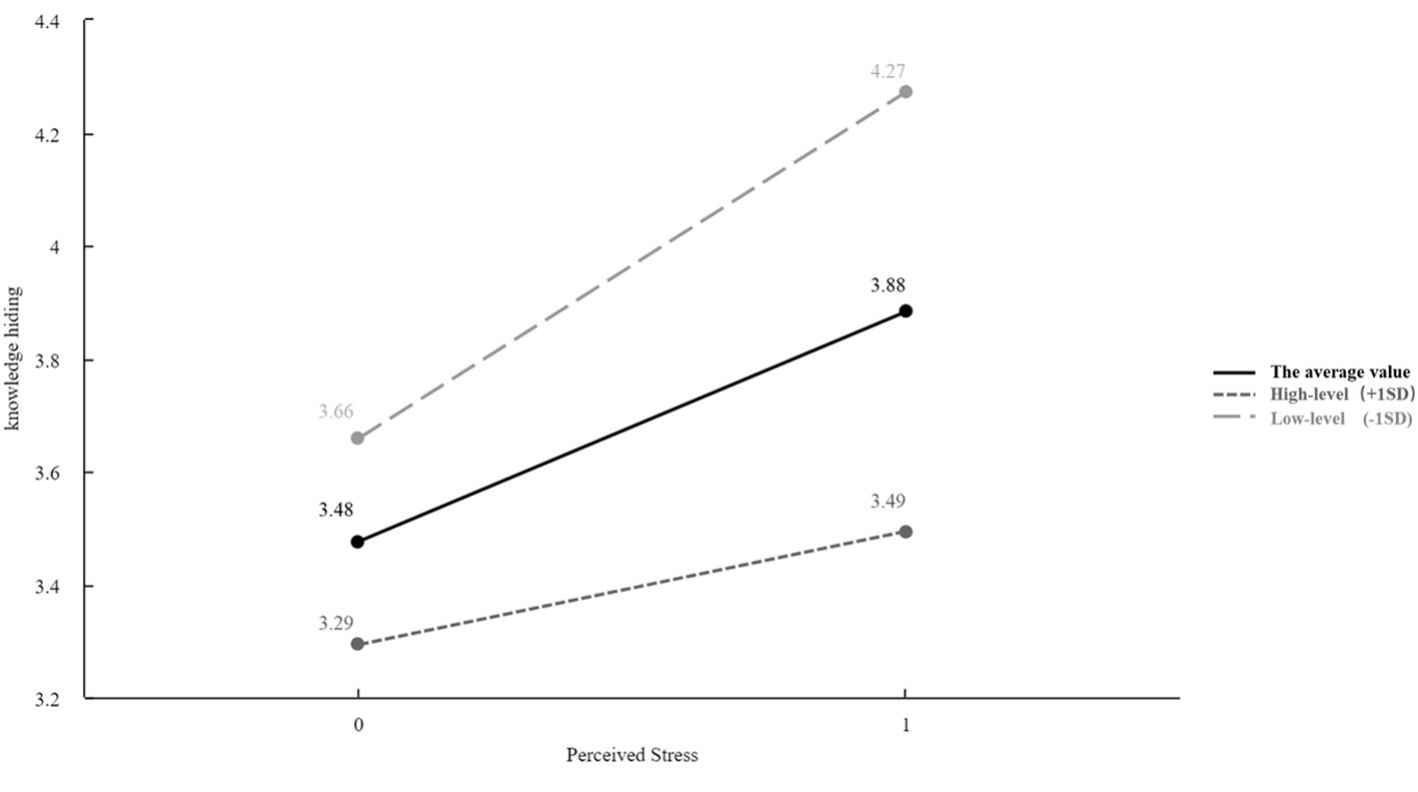
Simple slope plot.

Show in Table 3, the results of Model 2 showed that Workplace Ostracism (β = 0.198, *p* < 0.01) and Task complexity (β = 0.239, *p* < 0.01) both had significant positive effects on Perceived stress. When Knowledge hiding was entered as the dependent variable, the results of Model 4 revealed that both Workplace Ostracism (β = 0.180, *p* < 0.01) and Task complexity (β = 0.310, *p* < 0.01) significantly and positively influenced Knowledge hiding, thereby supporting Hypotheses 1 and 2. Model 5 demonstrated that Perceived stress positively predicted Knowledge hiding (β = 0.274, *p* < 0.01). After including Perceived stress in the regression, the coefficients of Workplace Ostracism and Task complexity decreased but remained significant, indicating that Perceived stress partially mediated these relationships. Following (MacKinnon et al. 2004), the bootstrap method was applied to test the indirect effects. Results indicated a significant mediating effect of Perceived stress on the relationship between Workplace Ostracism and Knowledge hiding (β = 0.054, *p* < 0.01), supporting Hypothesis 3. Similarly, the mediating effect of Perceived stress on the relationship between Task complexity and Knowledge hiding was significant (β = 0.066, *p* < 0.01), supporting Hypothesis 4. Model 6 introduced the interaction term between Perceived pay fairness and Perceived stress. The interaction term had a significant negative effect on Knowledge hiding (β = −0.261, *p* < 0.01), indicating that Perceived pay fairness moderated the relationship between Perceived stress and Knowledge hiding, thereby supporting Hypothesis 5. To further illustrate the moderation, a simple slope test was conducted. As shown in Figure 2, when Perceived pay fairness was low (M−1SD), the positive effect of Perceived stress on Knowledge hiding was stronger (β = 0.613, *p* < 0.001). Conversely, when Perceived pay fairness was high (M + 1SD), the effect was weaker (β = 0.201, *p* < 0.01), confirming the moderating role of Perceived pay fairness (Figure 2).

Finally, moderated mediation was tested using 5,000 bootstrap resamples, and the results are presented in Table 4. For both high and low levels of Perceived pay fairness, Workplace Ostracism had a significant indirect effect on Knowledge hiding through Perceived stress. The difference between high and low groups was significant (Δ = −0.074, 95% CI [−0.129, −0.020], excluding 0.), supporting Hypothesis 6a. Similarly, Task complexity had a significant indirect effect on Knowledge hiding through Perceived stress at both high and low levels of Perceived pay fairness, with a significant group difference (Δ = −0.083, 95% CI [−0.138, −0.028], excluding 0.). This finding supported Hypothesis 6b, indicating that Perceived pay fairness weakened the mediating role of Perceived stress in both paths.

**Table 4:** Results of moderated mediation analyses (Bootstrap, 5,000 resamples).

**Pathway**	**Moderator variable**	**Moderated indirect effect**		
		**Indirect effect (**β**)**	**SD**	**95% CI (LL, UL)**
Workplace ostracism → Perceived stress → Knowledge hiding	High (M + 1 SD	0.041	0.033	−0.023, 0.105
	Low (M−1 SD)	0.158	0.034	0.093, 0.225
	Difference (Δ)	−0.074^**^	0.028	−0.129,−0.020
Task complexity → Perceived stress → Knowledge hiding	High (M + 1 SD	0.019	0.033	−0.042, 0.085
	Low (M−1 SD)	0.151	0.031	0.090, 0.216
	Difference (Δ)	−0.083^**^	0.028	−0.138,−0.028

^**^p < 0.01.

## 5 Discussion

### 5.1 Conclusion

Grounded in conservation of resources theory, social exchange theory, and stress interaction theory, this study investigates the mechanisms and boundary conditions of Knowledge hiding among knowledge workers from the perspective of Perceived stress. The results indicate that Workplace Ostracism and Task complexity significantly and positively predict Knowledge hiding, with Perceived stress serving as a partial mediator. Furthermore, Perceived pay fairness negatively moderates both the direct relationship between Perceived stress and Knowledge hiding, as well as the indirect effects of Workplace Ostracism and Task complexity through Perceived stress. Specifically, high Perceived pay fairness weakens the positive impact of Perceived stress on Knowledge hiding and attenuates the mediating effects of Workplace Ostracism and Task complexity, whereas low Perceived pay fairness strengthens these relationships.

This study makes three main contributions. First, it enriches the literature on Knowledge hiding by revealing its formation mechanism from a stress-perception perspective, underscoring Perceived stress as a key explanatory mechanism. Second, it extends the application of conservation of resources theory and social exchange theory by uncovering the moderating role of Perceived pay fairness, thereby clarifying the boundary conditions under which workplace stressors affect Knowledge hiding. Third, it provides actionable insights for organizations: by improving compensation fairness, fostering supportive work environments, and alleviating Workplace Ostracism, managers can mitigate stress-induced Knowledge hiding, enhance trust, and promote knowledge sharing.

### 5.2 Managerial implications

Building on the above findings, this study proposes a set of strategic managerial interventions aimed at reducing Knowledge hiding and enhancing organizational knowledge sharing. These recommendations target four critical domains: workplace inclusion, task design, compensation fairness, and knowledge-sharing culture.

1. Fostering an inclusive work climate to mitigate Workplace Ostracism.

Workplace Ostracism was identified as a salient antecedent of Knowledge hiding. To address this issue, organizations should proactively meet employees' psychological and social needs by fostering a diverse, inclusive, and participatory climate. Recommended practices include establishing confidential employee voice channels (for example, anonymous suggestion boxes and encrypted feedback tools), integrating anonymous feedback into routine operations (such as periodic surveys or silent Q&A in team meetings), and ensuring equitable participation through quarterly role rotations or engagement tracking. Additionally, embedding “team integration” metrics into performance evaluations—through anonymous peer review—and offering access to professional counseling services can further enhance employees' sense of inclusion and psychological safety.

2. Optimizing task allocation and managing cognitive load

Misaligned task difficulty and role ambiguity can increase cognitive stress and incentivize knowledge withholding. Managers should ensure that Task complexity is appropriately matched with employee capabilities and available resources. Suggested interventions include workflow simplification supported by AI tools, the use of Task complexity assessment frameworks, and internal task scoring systems to improve task-person fit. Establishing transitional buffer periods (for example, 3 to 5 days of low-intensity work following high-pressure assignments) can facilitate recovery and skill development. For complex assignments, pre-task coordination meetings and the use of mind-mapping tools can help decompose tasks, clarify responsibilities, and ensure cross-departmental resource alignment.

3. Enhancing perceived pay fairness through transparent compensation systems

Perceived pay fairness moderates the relationship between stress and Knowledge hiding, making equitable pay systems a critical management lever. Organizations should enhance both procedural and distributive fairness through greater transparency in compensation evaluation. Recommended practices include developing digital platforms to visualize compensation structures and benchmark them against industry standards, establishing compensation calibration committees that publicly report quarterly findings, and applying factor comparison methods to determine base wages for knowledge-intensive roles. Tailored promotion and reward mechanisms should reinforce knowledge-sharing behaviors. Furthermore, instituting a tiered appeal and traceability mechanism—supported by error correction and routine employee feedback collection—can strengthen perceptions of legitimacy regarding the compensation system.

4. Institutionalizing a knowledge-sharing culture

Beyond structural adjustments, embedding knowledge sharing into the organizational culture is essential for fostering sustained behavioral change. Suggested initiatives include implementing incentive-based mentoring programs (for example, allowances for senior employees mentoring new hires for 6 m, evaluated via knowledge transfer outcomes) and adopting visual feedback tools such as shared contribution maps. Clearly defined key performance indicators (KPIs) and regular experience-sharing sessions (for example, brainstorming forums) can formalize expectations. Finally, establishing an error-tolerant protection system—including knowledge shelf-life exemptions, trial-and-error reimbursement mechanisms, and recognition for innovative initiatives—can create a psychologically safe space for experimentation and sharing.

Collectively, these managerial practices address both the motivational and structural drivers of Knowledge hiding, offering a multi-dimensional approach to cultivating a sustainable knowledge-sharing environment.

### 5.3 Limitations and future research directions

Firstly, this study's sample includes both domestic Chinese enterprises and some European and American multinational companies based in Beijing. While cultural factors have been somewhat controlled to minimize their influence on the findings, most Chinese companies still exhibit typical characteristics of “high power distance” and “collectivism.” These cultural factors may lead to differences in how Chinese employees perceive workplace Ostracism and pay fairness compared to employees from other cultural backgrounds. Specifically, Chinese employees may be more sensitive to workplace Ostracism and pay fairness, and their coping strategies in response to workplace stress may differ significantly from the more individualized and autonomous coping strategies commonly found in Western organizational contexts. Future research could expand the data collection to a broader range of regions, especially those with differing cultural backgrounds, in order to improve the external validity of the study and further test the generalizability of the findings.

Secondly, the sample in this study contains a relatively high proportion of younger employees under the age of 35. However, younger employees may respond differently to task complexity and workplace Ostracism compared to more experienced workers. While younger employees tend to possess strong learning and adaptability skills, their relative lack of experience makes them more susceptible to the pressures and challenges posed by task complexity and workplace Ostracism. This can lead to greater cognitive load and emotional fatigue, resulting in more frequent knowledge hiding behavior. In contrast, senior employees, with their greater work and interpersonal experience, may adopt different coping strategies based on their past experiences when facing complex tasks and workplace Ostracism. Future research could incorporate samples from different age groups to explore age-related differences in task complexity, stress perception, and knowledge hiding behavior, while also considering cross-cultural perspectives to assess the generalizability of the findings.

Finally, this study treats stress perception as a mediator and pay fairness perception as a moderator. However, knowledge hiding is the result of multiple interacting factors, and its triggering mechanisms are complex. Future research could employ fuzzy set qualitative comparative analysis (fsQCA) to introduce additional antecedents from a configurational perspective, thereby constructing a more comprehensive path model to enhance the explanatory power of the mechanisms underlying knowledge hiding behavior.

## Data Availability

The original contributions presented in the study are included in the article/ supplementary material, further inquiries can be directed to the corresponding author.
