# Correction: Why do knowledge workers choose knowledge hiding: the mediating role of perceived stress

**DOI:** 10.3389/fpsyg.2026.1792108

**Published:** 2026-02-16

**Authors:** Yina Bai, Yuxin Xiao

**Affiliations:** School of Business Administration, Liaoning Technical University, Huludao, China

**Keywords:** knowledge hiding, knowledge workers, perceived stress, task complexity, workplace ostracism

Several references were incorrect in the published article.

The reference for (Cook et al., 2024) was erroneously written as Cook, R. M., Blair, R., and Turpin, C. (2024). A longitudinal examination of the psychometric properties of the Perceived Stress Scale−4 using item response theory. *Stress Health* 40, 337–352. doi: 10.1002/smi.3468. It should be Cook, R. M., Wind, S. A., and Fye, H. J. (2024). A longitudinal examination of the psychometric properties of the English perceived stress scale-four (PSS-4) in mental health counsellors using item response theory. *Stress Health* 40:e3468. doi: 10.1002/smi.3468.

The reference for (Dong and Zhang, 2025) was erroneously written as Dong, Q., and Zhang, H. (2025). The double-edged effects of Workplace Ostracism on employee's knowledge sharing. J. *Northwest Norm. Univ*. 62, 133–144. doi: 10.1108/JKM-02-2024-0222. It should be Dong, Q., and Zhang, H. (2025). The double-edged effects of Workplace Ostracism on employee's knowledge sharing. *J. Northwest Norm. Univ*. 62, 133–144. doi: 10.16783/j.cnki.nwnus.2025.02.014.

The reference for (Fu et al., 2020) was erroneously written as Fu, Y., Peng, Q., and Zhong, X. (2020). Job autonomy, organizational hindrance perception, and employees' knowledge hiding. *Soft Sci*. 34, 131–135. doi: 10.1108/pr-03-2020-0133. It should be Fu, Y., Peng, Q., and Zhong, X. (2020). Job autonomy, organizational hindrance perception, and employees' knowledge hiding. *Soft Sci*. 34, 131–135.doi: 10.13956/j.ss.1001-8409.2020.06.21.

The reference for (Iftikhar and Ahmed., 2025) was erroneously written as Iftikhar, R., and Ahmed, S. (2025). Unveiling knowledge hiding in interorganizational projects: antecedents and performance implications. *J. Manag. Eng*. Advance online publication. 56:87569728251324479. doi: 10.1177/87569728251324479. It should be Iftikhar, R. (2025). Unveiling knowledge hiding in interorganizational projects. *Project Manag. J*. 56, 657–675. doi: 10.1177/87569728251324479.

The in-text citation was updated to (Iftikhar, 2025).

The reference for (Indhumathi and Thirumoorthi, 2025) was erroneously written as (2025). Effect of Workplace Ostracism on psychological distress, perceived stress, psychological detachment, and burnout among nurses in Tamil Nadu. *Multidiscip. Sci. J*. 7:e2025570. doi: 10.31893/multiscience.2025570. It should be Indhumathi, Thirumoorthi, and Bhuvaneswari. (2025). Effect of workplace ostracism on psychological distress, perceived stress, psychological detachment, and burnout among nurses in Tamil Nadu. *Multidiscip. Sci. J*. 7:2025570. doi: 10.31893/multiscience.2025570.

The in-text citation was updated to (Indhumathi et al., 2025).

The reference for (Kamilçelebi, 2024) was erroneously written as Kamilçelebi H. (2024). Fairness perceptions mediate the relationship between income comparisons and subjective well-being. *J. Econ. Psychol*. Advance online publication. 32, 2344–2348. doi: 10.1080/13504851.2024.2332584.

It should be Kamilçelebi, H., and Burger, M. J. (2025). Fairness perceptions mediate the relationship between income comparisons and subjective well-being: evidence from Türkiye. *Appl. Econ. Lett*. 32, 2344–2348. doi: 10.1080/13504851.2024.2332584.

The in-text citation was updated to (Kamilçelebi and Burger, 2025).

The reference for (Shen, 2025) was erroneously written as Shen Y. (2025). A meta-analysis of knowledge hiding behavior in organizations: antecedents, moderators, and outcomes. *J. Bus. Res*. 186:114963. doi: 10.1016/j.jbusres.2024.114963 It should be Shen, Y., Lythreatis, S., Singh, S. K., and Cooke, F. L. (2025). A meta-analysis of knowledge hiding behavior in organizations: Antecedents, consequences, and boundary conditions. *J. Bus. Res*. 186:114963. doi: 10.1016/j.jbusres.2024.114963.

The in-text citation was updated to (Shen et al., 2025).

The reference for (Srivastava and Dhir, 2024) was erroneously written as (Srivastava, S., and Dhir, S. (2024). Do workplace practices really matter? The role of ostracism and dehumanization for employees' psychological well-being. *Int. J. Organ. Anal*. Advance online publication. 32, 1574–1593. doi: 10.1108/IJOA-05-2023-3764. It should be Srivastava, S., and Dhir, S. (2024). Do workplace practices really matter? Role of ostracism and dehumanization at the workplace and psychological well-being of employees. *Int. J. Organ. Anal*. 32, 1574–1593. doi: 10.1108/IJOA-05-2023-3764.

The reference for (Xiao et al., 2023) was erroneously written as Xiao, T., He, J., and Chen, J. (2023). Psychometric validation of the Perceived Stress Scale (PSS-10) among Chinese healthcare workers. *BMJ Open* 13:e076372. doi: 10.1136/bmjopen-2023-076372 It should be Xiao, T., Zhu, F., Wang, D., Liu, X., Xi, S. J., and Yu, Y. (2023). Psychometric validation of the Perceived Stress Scale (PSS-10) among family caregivers of people with schizophrenia in China. *BMJ Open* 13:e076372. doi: 10.1136/bmjopen-2023-076372.

Several references have been replaced in the published article.

“Ahmad, S., Khan, A., and Raza, S. (2024). Task structure and knowledge transfer: the roles of task interdependence and knowledge complexity. *Behav. Inform. Technol*. 43, 2509–2525. doi: 10.1080/0144929X.2024.2383260” has been replaced with “Rasheed, M. I., and Pitafi, A. H. (2024). Task structure and knowledge transfer: leveraging employee agility performance in an ESM environment. *Behav. Inform. Technol*. 43, 2194–2208. doi: 10.1080/0144929X.2024.2383260”.

“Akram, A., Saeed, A., and Malik, M. (2024). Does Workplace Ostracism lead to workplace withdrawal? Evidence from a multi-method study. *J. Organ. Effect. People Perform*. 11, 873–896. doi: 10.1108/JOEPP-08-2023-0328” has been replaced with “Srivastava, S., Khan, M., Kumari, A., and Jain, A. K. (2024). Does workplace ostracism lead to workplace withdrawal? Testing the moderating-mediating effects of rumination and mindfulness in Indian hospitality industry. *J. Organ. Effect. People Perform*. 11, 873–891. doi: 10.1108/JOEPP-08-2023-0328”.

“Zhang, Y., Wang, L., and Li, J. (2025). A meta-analysis of workplace Ostracism on employee work outcomes. *Front. Psychol*. 16:1280074. doi: 10.3389/fpsyg.2025.1280074” has been replaced with “Li, Z. D., and Li, W. Y. (2025). A meta-analysis of workplace exclusion on employee work behavior in the Chinese context. *Front. Psychol*. 16:1280074. doi: 10.3389/fpsyg.2025.1280074.”

The original version of this article has been updated.

